# Metabolic drift in the aging nervous system is reflected in human cerebrospinal fluid

**DOI:** 10.1038/s41598-021-97491-1

**Published:** 2021-09-22

**Authors:** Kristian Peters, Stephanie Herman, Payam Emami Khoonsari, Joachim Burman, Steffen Neumann, Kim Kultima

**Affiliations:** 1 Department of Medical Sciences, Clinical Chemistry, Uppsala University, 751 85 Uppsala, Sweden; 2 Leibniz Institute of Plant Biochemistry, Bioinformatics and Scientific Data, Weinberg 3, 06120 Halle (Saale), Germany; 3 Department of Biochemistry and Biophysics, National Bioinformatics Infrastructure Sweden, Science for Life Laboratory, Stockholm University, Stockholm, Sweden; 4 Department of Neuroscience, Neurology, Uppsala University, 751 85 Uppsala, Sweden

**Keywords:** Metabolomics, Ageing

## Abstract

Chronic diseases affecting the central nervous system (CNS) like Alzheimer’s or Parkinson’s disease typically develop with advanced chronological age. Yet, aging at the metabolic level has been explored only sporadically in humans using biofluids in close proximity to the CNS such as the cerebrospinal fluid (CSF). We have used an untargeted liquid chromatography high-resolution mass spectrometry (LC-HRMS) based metabolomics approach to measure the levels of metabolites in the CSF of non-neurological control subjects in the age of 20 up to 74. Using a random forest-based feature selection strategy, we extracted 69 features that were strongly related to age (*p*_age_ < 0.001, r_age_ = 0.762, R^2^_Boruta age_ = 0.764). Combining an in-house library of known substances with in silico chemical classification and functional semantic annotation we successfully assigned putative annotations to 59 out of the 69 CSF metabolites. We found alterations in metabolites related to the Cytochrome P450 system, perturbations in the tryptophan and kynurenine pathways, metabolites associated with cellular energy (NAD+, ADP), mitochondrial and ribosomal metabolisms, neurological dysfunction, and an increase of adverse microbial metabolites. Taken together our results point at a key role for metabolites found in CSF related to the Cytochrome P450 system as most often associated with metabolic aging.

## Introduction

Advanced chronological age is considered as one of the most important risk factors for various kinds of diseases, especially chronic diseases and neurodegenerative diseases like Alzheimer's and Parkinson’s disease. Aging in humans is the combined result of genetic and non-genetic predispositions and extrinsic factors including lifestyle (i.e., physical activity, occupational stress, food diet) and environmental exposures (i.e., exposure to chemicals, pesticides and sun)^1,2^. As a result, human individuals age differently and physiological functions that determine health deteriorate highly variably among individuals^2^. Nevertheless, several attempts have been made to define a biological age using a multitude of physiological and metabolic traits^3^.

The metabolome is defined as a snapshot of the sum of small molecules (molecular weight between, i.e., 60–1500 Da) that characterizes a biological system. It changes considerably with chronological age and in human blood and urine the physiological changes that occur with aging have been demonstrated^3–10^. Recently, advances have been made to link advancing age with increasing chronic disease risk using metabolomics techniques to account for the physiological variation in the trajectory of human aging for various types of chronic and neurodegenerative diseases^3,11–14^. This is being achieved by determining multiple biomarkers that are highly correlated with age and are known to play a role with certain types of diseases. While this has previously been assessed in blood^13^ and plasma^3^, limited work has so far only explored chronological aging in cerebrospinal fluid (CSF) in children^15^, HIV positive patients^16^, or targeting a limited number of metabolites^17,18^ . The cerebrospinal fluid provides mechanical protection to the brain and prevents damage due to collision between the brain and skull. The CSF also influences metabolic homeostasis of the central nervous system (CNS) by maintaining the electrolytic environment and systemic acid base balance^19^. The CSF also serves as a medium for the supply of nutrients to neuronal cells and functions as a lymphatic system for removal of degradation products of cellular metabolism and transports hormones, neurotransmitters and other releasing factors throughout the CNS^19^. Moreover, the CSF serves as an important diagnostic tool in the evaluation of diseases affecting the CNS which typically develop with chronological aging^20^.

In this study, we aim to identify the metabolite features that show the strongest association with aging and to provide a systemic view on aging by identifying major pathways and metabolic systems involved. Using high-resolution mass spectrometry, we investigated the CSF metabolic content in a cohort of 41 subjects, ranging from the age of 20 up to 74, that do not suffer from any neurodegenerative disorders. To provide a systemic view on changes that occur with aging, we perform in silico classification and combine it with functional semantic annotation. This approach can give insight into mechanistic processes on how the CSF differs from blood or serum, and which types of metabolites and associated molecular pathways and systems are affected by aging at a global level.

## Results

**Figure 1:**
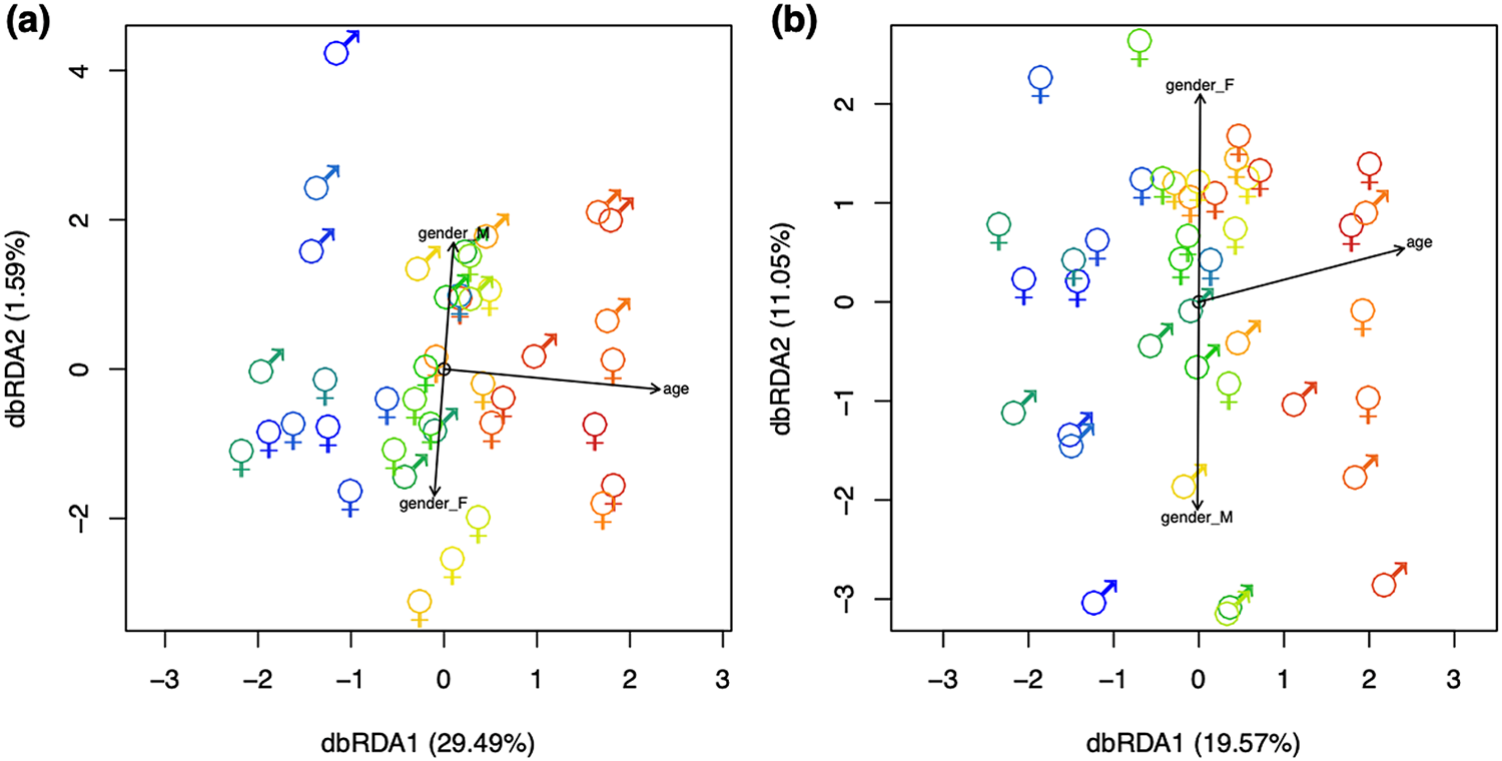
Distance-based redundancy analysis (dbRDA) constrained to the factors age and gender. The age of the subjects is color-coded, ranging from blue color (young subjects starting from the age of 20) to red color (older subjects with the oldest one being 74), whereas gender is displayed by the gender symbols. **(a)** The multivariate regression analysis on all 1841 metabolite features demonstrated a strong effect of aging on the direction of the x-axis and a weaker effect of gender on the direction of the y-axis. The directions of the factors age and gender were projected on the plot as arrows. Gender was largely orthogonal from age. **(b)** Random forest-based feature selection resulted in 69 features that were strongly related to age.

The CSF metabolome of 41 non-neurological control subjects between the age of 20 up to 74 was investigated using untargeted high-resolution mass spectrometry. A total of 1169 metabolite features in positive mode and 672 metabolite features in negative mode were successfully quantified and matched across at least 75% of the samples. To analyze the overall effect of aging, distance-based ReDundancy Analysis (dbRDA)^21^ was performed. This type of test allows to assess the impact and direction of aging in the data using multivariate analysis. Permutation tests on the dbRDA model showed overall strong effects of age and a less pronounced but significant effect of gender in the data (Empirical *p*-values: *p*_age_ < 0.001, *p*_gender_ < 0.001. Goodness of fit statistics: Squared correlation coefficients: r_age_ = 0.618, r_gender_ = 0.582), which were largely independent of each other (Fig. 1a). Furthermore, gender explained less total variance (≅ 1.59%, which was largely linearly corresponding to the y-axis) than age (≅ 29.49%, largely corresponding to the x-axis) (Fig. 1a).

**Figure 2:**
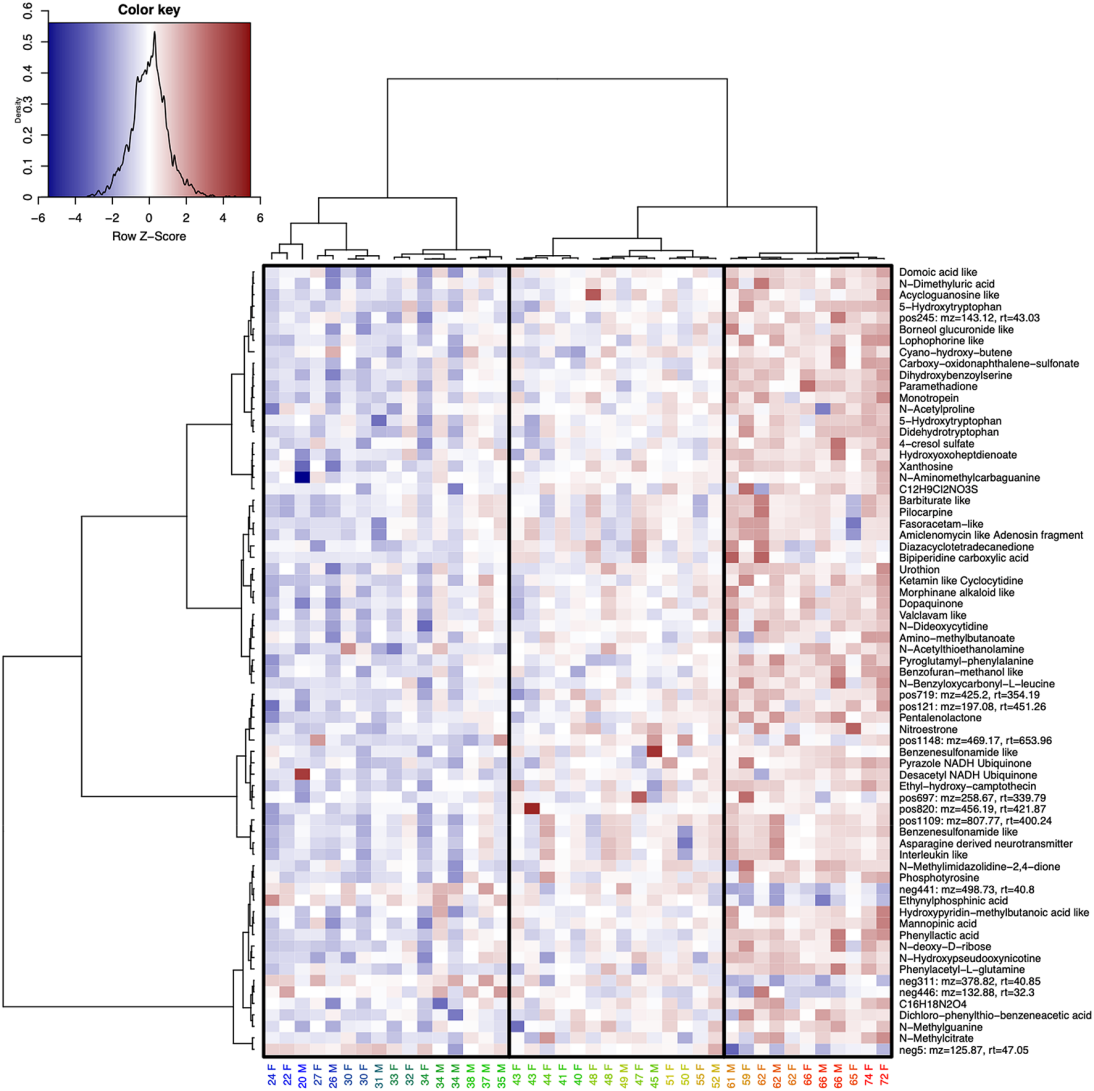
Heatmap showing selected metabolite features clustered in rows and the samples clustered by age in columns. The last letter of the sample name indicates the gender of the subject (M: Male, F: Female). A red color indicates a higher concentration of a metabolite feature and a blue color indicates a lower concentration. Three major groups of subjects were found, “Young age” (ages 20–37), “Middle age” (ages 40–59) and “Old age” (ages 59–74), of which each could be broken down into several subgroups.

Next, we investigated which metabolite features showed the strongest association with age and gender. Using a random forest-based feature selection strategy utilizing the Boruta algorithm^22^, we extracted 69 features that were strongly related to age (Fig. 1b) and 41 features that were related to gender (Fig. S4). Selected metabolite features showed a strong relationship and a good representation of the predicted vs. actual models for age (*p*_age_ < 0.001, r_age_ = 0.762, R^2^_Boruta age_ = 0.764) and gender (*p*_gender_ < 0.001, r_gender_ = 0.549, R^2^_Boruta gender_ = 0.469). Also, in this limited subset of features, age and gender were largely orthogonal. As the selected gender-associated features were not overlapping with the age-associated features, gender was not investigated any further (Fig. 2, Fig. S4).

Using hierarchical clustering on the selected features we found three major groups of subjects: “Young age” (ages ranging from 20 to 37), “Middle aged” (ages 40 to 59) and “Old age” (ages 59 to 74) (Fig. 2). We applied Dynamic Time Warping (DTW) to the data and it showed that most relationships with aging were linear (Fig. S1). Thus, the relationship between the metabolite abundance and age were tested with linear regression (Fig. S2). Looking at the age-dependencies, most of the selected features were increasing with aging, whereas only five were significantly decreasing (Ethynylphosphinic acid (ned227) and four unknown compounds (neg5, neg446, neg311, neg441) in Table 1, Fig. 2, S2).

**Table 1:** List of metabolite features that showed the strongest relationship with aging in human CSF.

ID	*m/z*	*rt *(s)	*p*-value	Putative name	Corresponding identifiers	Putative functional/biological roles	MSI level
pos1168	98.05	37.58	0.040	Cyano-hydroxy-butene	HMDB: HMDB0031339, PUBCHEM: 91586	Food source, amino acid metabolism, edema, necrosis, pancreatic diseases	2
pos207	120.04	265.57	0.026	*S*-Acetylthioethanolamine	KEGG: C03537, PUBCHEM: 28643, CHEBI: 17853	Microbial metabolism	2
pos245	143.11	43.03	0.024			Unknown	-
pos175	157.04	39.88	0.018	Hydroxyoxoheptdienoate	KEGG: C06210, PUBCHEM: 9776837, CHEBI: 1132	Microbial metabolism	2
pos726	158.08	145.80	0.019	*N*-Acetylproline	HMDB: HMDB0094701, PUBCHEM: 66141, CHEBI: 21560	Proline metabolism, nervous system, pulmonary disease, chronic obstructive, inflammation, colorectal cancer	1
pos171	158.08	58.84	0.022	Paramethadione	HMDB: HMDB0014755, KEGG: C07411, PUBCHEM: 8280, CHEBI: 7921	Cytochrome P450, NADPH-related, monooxygenase activity, ion channels, antiepileptic	2
pos441	166.07	51.89	0.010	*N*-Methylguanine	KEGG: C02242, HMDB: HMDB0001566, PUBCHEM: 70315, 135398679, CHEBI: 2274	Immune system, purine metabolism, colorectal cancer, catalytic activity, microbial ribosomal role	1
pos1046	180.08	257.27	0.021	7-Aminomethyl-7-carbaguanine	KEGG: C16675, CHEBI: 45126, PUBCHEM: 135398563, HMDB: HMDB0011690	NAD^+^-related, pyrimidine metabolism, tRNA-related, microbial metabolism, folate biosynthesis	2
pos266	195.07	72.04	0.014	*N*-Dimethyluric acid	PUBCHEM: 91,611, CHEBI: 68,449, HMDB: HMDB0011103, KEGG: C16356	Caffeine metabolism, Cytochrome P450, xanthine dehydrogenase/oxygenase, colorectal cancer	2
pos170	195.11	319.97	0.026	*N*-Hydroxypseudooxynicotine	KEGG: C01297, PUBCHEM: 439476, CHEBI: 37754, HMDB: HMDB0240264	NAD^+^-related, nicotinamide metabolism, microbial metabolism	2
pos537	196.06	317.68	0.020	Dopaquinone	HMDB: HMDB0001229, KEGG: C00822, PUBCHEM: 439316, CHEBI: 16852	Neurodegeneration, Parkinson’s disease, melanin-precursor, several other metabolic disorders, tyrosine metabolism, DOPA	2
pos121	197.08	451.26	0.024			Unknown	-
pos1075	197.13	421.64	0.030	Alpha amino acid	CHEMONT: C0002404		4
pos1147	197.13	413.42	0.029	C10H16N2O2		Unknown	-
pos477	203.08	63.90	0.023	α,β-Didehydrotryptophan	KEGG: C06732, CHEBI: 15802, PUBCHEM: 5280990	Tryptophan and kynurenine pathway	2
pos102	209.13	340.59	0.037	Pilocarpine	KEGG: C07474, HMDB: HMDB0015217, CHEBI: 8207, PUBCHEM: 5910	Vascular related, inflammation, histidine and purine pathways, nervous system: acetylcholine-receptor, epilepsy, Cytochrome P450, acetylcholine receptor	3
pos324	212.10	71.87	0.017	*N,N*-Dideoxycytidine	KEGG: C07207, CHEBI: 10101, PUBCHEM: 24066, HMDB: HMDB0015078	Immune system, anti-viral drug, RNA/DNA polymerase related	2
pos1061	213.16	419.33	0.027	Bipiperidine carboxylic acid	CHEBI: 80763, KEGG: C16836, PUBCHEM: 11367848, HMDB: HMDB0060336	Lipid transport and metabolism, liver detoxification	2
pos568	221.09	63.86	0.022	5-Hydroxy-l-tryptophan	KEGG: C00643, ChEBI: 17780, PUBCHEM: 439280, HMDB: HMDB0000472	Tryptophan and kynurenine pathway, neurodegeneration, neurological, neurotransmitter-precursor, DOPA	1
pos296	226.08	44.75	0.018	Acycloguanosine/hypoxanthine	CHEMONT: C0000246	DNA-synthesis, neurological, NAD^+^-related, poisoning, uremia (waste product degeneration), colorectal cancer	3
pos180	227.14	340.68	0.036	Barbiturate/pyrimidone	CHEMONT: C0000291	Neurological: ion channels, Cytochrome P450, inflammation	3
pos99	227.17	488.20	0.037	Diazacyclotetradecanedione	KEGG: C04277, CHEBI: 16968, PUBCHEM: 16, HMDB: HMDB0033567	Microbial metabolism, caprolactam degradation	3
pos75	238.11	417.12	0.021	Cyclocytidine/alkyl aryl ether	CHEMONT: C0000128	Neurological: ion channels	3
pos697	258.66	339.78	0.012			Unknown	-
pos588	266.14	338.12	0.042	*N*-Benzyloxycarbonyl-l-leucine	KEGG: C04335, CHEBI: 28282, PUBCHEM: 74840	Leucine metabolism, uremia (waste product degeneration)	2
pos285	277.12	425.06	0.024	Pentalenolactone	KEGG: C20407, PUBCHEM: 24199350, CHEBI: 70816	Microbial metabolism	2
pos1054	580.06	408.41	0.013	Mannopinic acid	PUBCHEM: 126642	Microbial metabolism, immune system	2
pos328	312.15	330.58	0.016	Morphinan	CHEMONT: C0000058	Neurological, neurotransmitter (G protein-coupled receptors)	3
pos865	316.16	461.10	0.026	Nitroestrone	CHEBI: 79864, PUBCHEM: 233497, KEGG: C15362	Hormone	2
pos361	326.04	340.04	0.021	Urothion	HMDB: HMDB0002377, PUBCHEM: 135804811, CHEBI: 50152	Uremia (waste product degeneration)	2
pos513	330.17	330.70	0.016			Unknown	-
pos927	393.16	346.68	0.021	Ethyl-hydroxy-camptothecin	KEGG: C11173, CHEBI: 94969, PUBCHEM: 104842, HMDB: HMDB0060510	Cancer, irinotecan metabolism, Waste product degeneration	2
pos928	415.22	361.01	0.015	Deacetylvindoline	PUBCHEM: 260534, KEGG: C01091, CHEBI: 18362	Microbial metabolism, food source, indole alkaloid metabolism	3
pos887	422.21	310.11	0.039	Fenpyroximate	KEGG: C11098, PUBCHEM: 9576412, CHEBI: 5011	NAD^+^-related, ubiquinone pathway, Parkinson disease, microbial metabolism	2
pos719	425.20	354.18	0.019			Unknown	-
pos820	456.19	421.86	0.019			Unknown	-
pos1148	469.16	653.96	0.018			Unknown	-
pos1035	742.33	401.81	0.018	Benzenesulfonamide analog	PUBCHEM: 5278358, CHEMONT: C0000031	Microbial metabolism, anti-viral (HIV protease inhibitor)	4
pos1064	745.35	409.10	0.038	Cyclic peptide	PUBCHEM: 74223411, CHEMONT: C0001995	Inflammation, viral related	4
pos1033	745.51	409.00	0.037	Asparagine and derivatives	PUBCHEM: 189479, CHEMONT: C0004312	Anti-inflammation, asparagine derived neurotransmitter	4
pos1037	790.15	414.75	0.049	Benzenesulfonamide analog	PUBCHEM: 123600247, CHEMONT: C0000031	Inflammation, microbial metabolism	4
pos1109	807.77	400.24	0.056			Unknown	-
*neg5*	*125.87*	*47.04*	*− 0.005*			Unknown	-
*neg446*	*132.88*	*32.29*	*− 0.002*			Unknown	-
neg553	133.04	49.06	0.038	2-Deoxy-d-ribose	CHEBI: 28816, HMDB: HMDB0003224, PUBCHEM: 5460005, KEGG: C01801	DANN, ATP, cancer, pentose phosphate pathway	1
neg436	147.04	351.62	0.023	*N*-Nonatriene-*N*-diol	PUBCHEM: 101408974, CHEMONT: C0001334	Microbial metabolism	2
neg230	165.05	351.48	0.019	*N,N*-Dihydroxyphenyllactic acid	KEGG: C01207, PUBCHEM: 439435, HMDB: HMDB0003503	Food source, cerebral ischemia, cardiovascular diseases, hormone synthesis	2
*neg227*	*184.94*	*40.23*	*− 0.012*	Ethynylphosphinic acid	PUBCHEM: 118,074,783		2
neg301	187.00	299.82	0.016	*P*-cresol sulfate	PUBCHEM: 4615423, HMDB: HMDB0011635, CHEBI: 82914, KEGG: C01468	Neurodegeneratiion (multiple sclerosis), Crohn’s disease, cardiovascular diseases, microbial metabolism, uremia, colorectal cancer	2
neg273	205.03	54.29	0.014	*N*-methylcitrate	KEGG: C02225, CHEBI: 10860, PUBCHEM: 12898022, HMDB: HMDB0000379	Crohn’s disease, immune system, Vitamin B12 deficiency, microbial metabolism, propanoate metabolism	2
neg466	215.13	81.75	0.018	Valine and derivatives	PUBCHEM: 54064935, CHEMONT: C0004310	microbial metabolism	4
neg461	219.07	63.12	0.028	*N*-Hydroxy-l-tryptophan	KEGG: C19716, PUBCHEM: 23657839, CHEBI: 47992, HMDB: HMDB0000472	Neurological, neurodegenerative (Parkinson’s disease), neurotransmitter, DOPA, AADC deficiency, inflammation, tryptophan metabolism, cancer	1
neg306	225.08	157.82	0.016	*N,N*-Dihydroxy-alpha-methylstilbene	PUBCHEM: 69933604, CHEMONT: C0002279	Microbial metabolism	2
neg334	236.09	409.59	0.022	Tetrahydroisoquinoline	PUBCHEM: 442315, CHEMONT: C0002955	Microbial metabolism	2
neg417	240.05	306.99	0.023	*N,N*-Dihydroxybenzoylserine	KEGG: C04204, CHEBI: 17455, PUBCHEM: 151483	Microbial metabolism, microbial ribosomal role	2
neg214	260.02	50.71	0.014	Phosphotyrosine	KEGG: C06501, PUBCHEM: 30819, CHEBI: 37788, HMDB: HMDB0006049	Food source, microbial metabolism, accumulation of waste products, tyrosine and phenylalanine metabolism, carcinogenesis	2
neg279	263.10	312.55	0.048	Phenylacetyl-l-glutamine	KEGG: C04148, PUBCHEM: 92258, CHEBI: 17884, HMDB: HMDB0006344	Microbial metabolism, phenylacetate metabolism, uremia, cancer, neurodegeneration,, immune system	2
neg474	264.98	351.54	0.022	Naphthalene sulfonic acid	PUBCHEM: 142667787, CHEMONT: C0003599	Microbial metabolism	3
neg523	275.10	417.93	0.032	Pyroglutamyl-phenylalanine	PUBCHEM: 7408331	Cancer, phenylalanine pathway, microbial metabolism, neurodegeneration, immune system	2
neg304	283.06	92.23	0.027	Xanthosine	KEGG: C01762, CHEBI: 18107, PUBCHEM: 64959, HMDB: HMDB0000299	Purine metabolism, leukaemia, colorectal cancer, uremia (waste product degeneration), Crohn’s disease, immune deficiency, NAD^+^-related, nucleotide binding	2
neg540	302.05	58.02	0.015	Benzo-1,2,3-triazine	PUBCHEM: 142469963, CHEMONT: C0004659		3
neg407	310.11	247.37	0.017	Kainoid	PUBCHEM: 5282253, CHEMONT: C0001801	Neurotoxin, food source	3
neg207	310.96	47.31	0.014	Benzeneacetic acid analog	PUBCHEM: 826412, CHEMONT: C0003864	Inflammation	4
neg336	315.95	92.45	0.011	C12H9Cl2NO3S			-
neg291	328.15	318.92	0.015	Terpene glycoside	PUBCHEM: 191872, CHEMONT: C0002049	Isoborneol glucuronide metabolism	4
neg176	332.95	46.51	0.011	Hydantoin	CHEMONT: C0002273	Microbial metabolism, inflammation, non-opioid receptor related, DNA polymerase related, cancer	3
*neg311*	*378.81*	*40.84*	− 0.005				-
neg386	389.09	444.39	0.028	Iridoid *O*-glycoside	CHEMONT: C0004081	Inflammation	3
*neg441*	*498.72*	*40.79*	− 0.005				-

The columns show the feature ID, mass-to-charge ratio including charge (*m/z*) (Da), retention time (s), the *p*-value of the linear regression model (positive values indicate an increase with progressing age, negative values indicate a decrease, *p* < 0.05 are considered to be significant), the annotated putative name, identifiers in the corresponding internet databases HMDB, KEGG, PubChem, and ChEBI, the manually curated putative functional and biological roles and MSI levels. We confirmed 4 compounds with our in-house library (conforming to MSI level 1 annotation according to^58^), we matched 32 compounds in public libraries (conforming to MSI level 2 annotation), we annotated 12 compounds using SIRIUS and MetFrag (MSI level 3 annotation) and 8 compounds using our classification framework (MSI level 4 annotation). Three compounds were annotated with a sum formula and 10 metabolite features could not be annotated.

Significantly decreasing features are illustrated in italic.

### Identification and chemical classification of selected features

**Figure 3:**
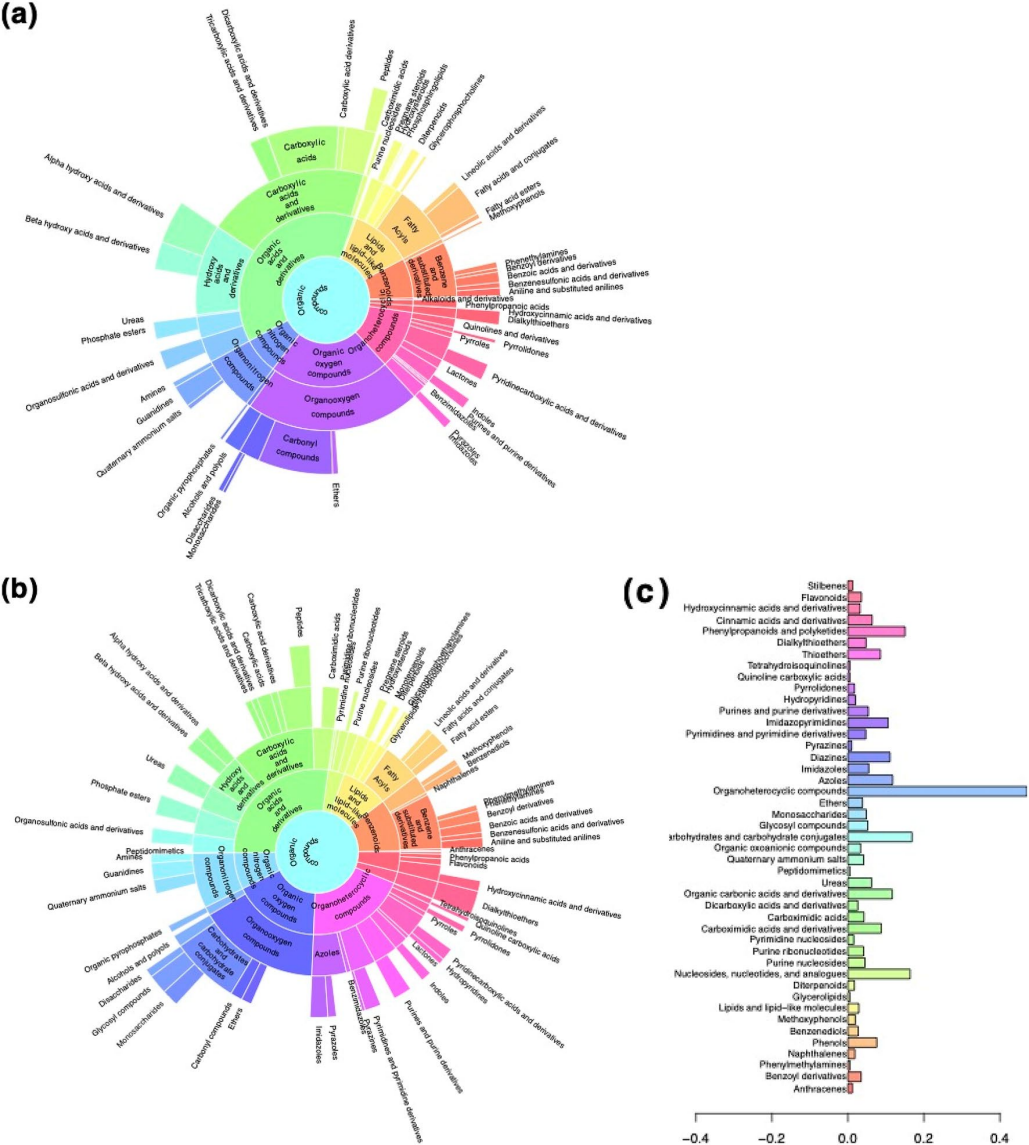
Plots showing the difference in the chemical classification of **(a)** the entire set of MS2 DDA spectra found in CSF samples shown as a sunburst plot, **(b)** selected metabolite features that were related to aging (listed in Table 1) in human CSF shown as a sunburst plot, **(c)** differences between the two sunburst plots calculated with the Fisher’s exact test (p < 0.005). Shown are the 45 top-most enriched compound classes. The sunburst plots represent the hierarchy and richness of compound classes. Starting in the center with organic compounds and towards the edges the more specific subclasses are shown. The width and color of each (sub)class correspond to the number of chemical entities assigned to this class.

Identification of CSF metabolites that showed the strongest relationship with age was carried out with the tools SIRIUS and MetFrag from the msPurity workflow suite^23–25^. Using this approach, we assigned putative annotations to 59 out of the 69 selected features, resulting in a large diversity of different metabolites (Table 1, Fig. 3a). To further investigate whether specific compound classes were more often associated with aging, we applied in silico classification and assigned chemical classes to 49 out of 69 features. The represented compound classes showed a similarly large diversity, where the most entities related to aging were found in the superclasses organic acids and derivatives, organoheterocyclic compounds, organic oxygen compounds, benzenoids and lipids and lipid-like molecules (Fig. 3b, Table S1).

When comparing the classification of the 69 selected features (Fig. 3b) with the entire set of MS2 DDA spectra found in the CSF samples (Fig. 3a), enriched compound classes (Fisher’s exact test, p < 0.005) related to aging were organoheterocyclic compounds (like ureas, imidazoles, diazines, azoles), phenylpropanoids and polyketides (flavonoids, cinnamic acids), carbohydrates (especially monosaccharides), organic carbonic acids (like ethers), nucleosides and nucleotides (including purines and pyrimidines) and various phenols (Fig. 3c). Organic oxygen compounds were less often associated with aging when compared to the entire set.

### Classification of functional roles

**Figure 4:**
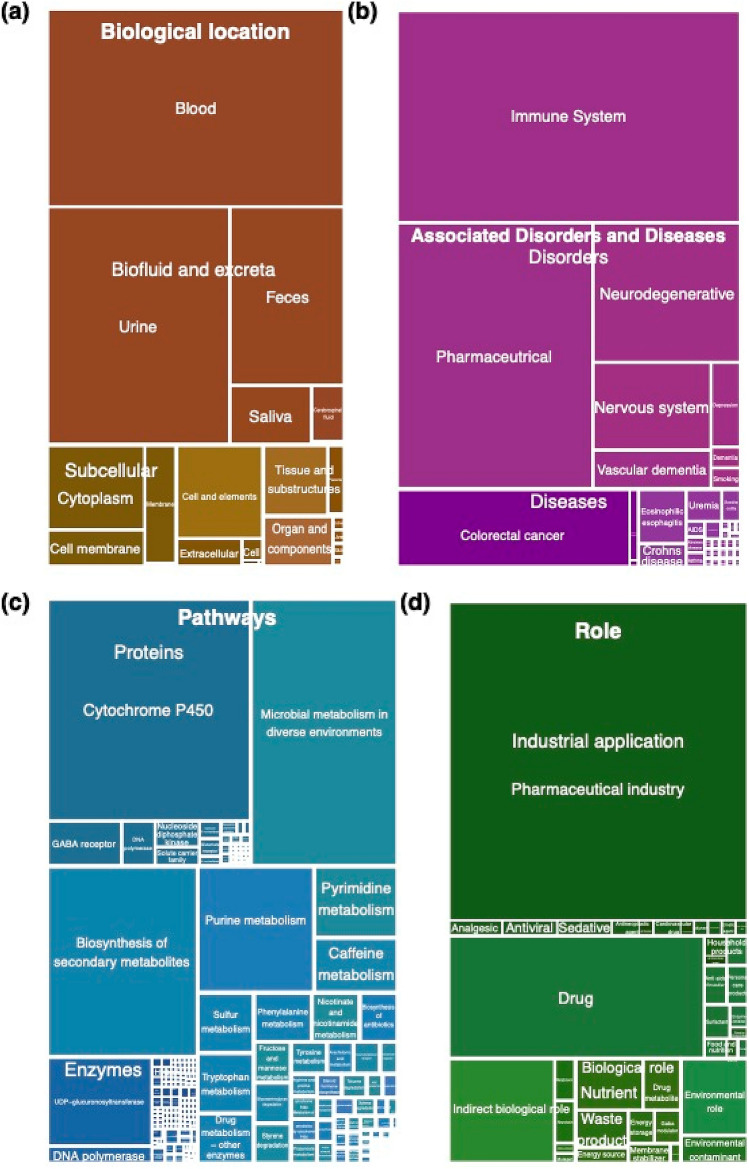
Treemap plot showing the location, functional role and associations of the 69 CSF metabolites that were strongly associated to aging in humans as annotated in HMDB. **(a)** Treemap for the concepts on “Biological Location”. **(b)** Treemap for the concepts on “Disorders and Diseases”. **(c)** Treemap for the classes on “Pathways”. **(d)** Treemap for the classes on “Role”. The four treemaps are plotted equally to each other. Classes are emphasized by color. The areas correspond to the number of chemical entities assigned to this class. The label size represents the ontology level where large labels are shown for the superclass and smaller labels for subclasses and lower ontology levels. All figures and underlying data are available as PDF in the Supplement in Zenodo (doi:10.5281/zenodo.5082928).

To investigate whether there were functional roles that were more often associated with aging, we combined the information from the chemical identities of the annotated features with the semantic information extracted from public resources, including metabolic pathways, connected proteins and enzymes, biological locations and roles, and associated disorders and diseases. The results were filtered and grouped into a total of 374 ontology terms (Fig. 4). The more often a specific ontology term was associated with our identified set of metabolites, the larger the area is in the treemap. The most frequently annotated biological locations were blood, urine and faeces, whereas only a few of the identified features had been previously detected in CSF and reported as such in HMDB. Only 5-hydroxy-L-tryptophan, mannopinic acid, N-methylcitrate, and phenylacetyl-L-glutamine had previously been reported in HMDB as detected in CSF.

We found that those metabolites were most often associated with aging that were related to the cytochrome P450 system, microbial metabolism, the immune system and gamma-aminobutyric acid (GABA). We also observed specific metabolic pathways that were associated with aging: purine-, pyrimidine-, caffeine-, tryptophan- and phenylalanine metabolisms, and nicotinamide (NAD+) related substances and derived enzymes (Fig. 4a–c). Many metabolites have been associated with neurodegenerative disorders and diseases affecting the CNS. The most pronounced non-neurological disease found was colorectal cancer (Fig. 4b). Besides relationships to pharmaceutical products and drug metabolism, we found biological roles to be related to cellular waste and toxic products and cellular energy- and membrane-metabolism (Fig. 4d).

## Discussion

Aging is the single greatest risk factor associated with numerous diseases including cancer, metabolic and cardiac disorders and diseases affecting the CNS such as Alzheimer's disease. As the population is continuously getting older, age-related or age-mediated diseases will be an increasing problem and will become a greater burden to healthcare systems worldwide.

To get a better understanding of the naturally occurring aging process in the CNS of humans, we have analyzed the concentrations of metabolites found in the human CSF from 41 non-neurological control subjects in the age span of 20 to 74 years of age using high-resolution mass spectrometry. Using a random forest-based variable selection strategy we found 69 features that showed strong age dependence. Using hierarchical clustering, the subjects could be divided into three distinct age groups: young, middle-aged and elderly subjects. This is in line with previous findings which studied the aging murine brain, where a metabolic drift was found as a result of aging in different parts of the brain^26^. Here, we demonstrate that metabolic drifts occurring in the aging human CNS can be reflected in a localization of CSF. Metabolic studies of blood collections (serum or plasma)^3–9^ and urine^10^ have previously demonstrated that metabolite levels are influenced by aging. Aging has been assessed in relation to the CSF metabolome, where HIV positive patients were compared with HIV negative controls and the results suggested that the HIV positive patients exhibited accelerated aging, since their CSF metabolomes overlapped with the controls of advanced age^16^. A different study performed metabolic screening in CSF profiles of juveniles and compared to patients with known neurometabolic disorders. They found a subset of 17 metabolites to be different between age groups in children up to the age of fifteen^15^. They found many amino acids like L-glutamine, L-leucine, L-phenylalanine and metabolites related to tryptophan metabolism such as L-tryptophan and 5-hydroxy-L-tryptophan) and purine and pyrimidine metabolism (e.g., hypoxanthine), to show different concentrations between age-groups, which to a large extent agrees with findings made herein in adults. Furthermore, we were able to relate metabolites to specific intermediary components of pathways in humans as part of leucine, tyrosine, phenylalanine, tryptophan and kynurenine pathways. For most of the 69 metabolites that showed the strongest relationship with age we found a linear relationship between the metabolite concentration and age. Only five out of the 69 selected metabolites showed decreasing levels with aging.

To get an overview of which chemical classes, biochemical pathways and compartments were affected by aging, semantic annotations for the 59 identified metabolites were extracted from public databases. The results showed that the metabolites comprised a large diversity of compound classes, including organic acids and derivatives, organic oxygen compounds, benzenoids, lipids and lipid-like molecules. We found a statistically significant enrichment of organoheterocyclic compounds, which is a high-level classification of compounds containing a ring with at least one carbon atom and one non-carbon atom, thus constituting a large variety of molecules. Furthermore, the dominantly represented locations previously annotated for these compounds included blood, urine and faeces. CSF was less evident probably due to it being less characterized and annotated in public databases. Previous findings in blood have demonstrated that steroid lipids, amino acids and higher levels of fatty acids are metabolites showing the strongest association with aging^3,8^, whereas the results herein display a much more diverse group of metabolites. High-level analysis of the metabolites clearly demonstrated that the cytochrome P450 system, the immune system as well as substances related to the microbial system were associated most often with aging. We also observed a significant enrichment of nucleosides/nucleotides, metabolites typically involved in the purine-, pyrimidine- and NAD^+^ energy related-metabolism, as well as caffeine-, tryptophan- and phenylalanine metabolisms. Multiple metabolites have also been associated with disorders of the CNS and neurodegeneration.

### Microbial-associated metabolites

We found several metabolites annotated with microbial origin in CSF which were increasing with age (Table 1). Metabolites derived from the gut microbiome can be transported via blood into CSF through the gut-brain axis (GBA)^27^. Tryptophan-derived metabolites play a key role in the immune response as tryptophan is metabolized into kynurenine, tryptamine and indole. Tryptophan-derived tryptamine and serotonin have also been described to modulate degradation pathways of microbial products. The microbial-associated metabolites in CSF that we found to be increased with age may indicate an increasingly less effective catabolism.

### Cytochrome P450 system

The cytochrome P450 system plays an important role in cellular metabolism and hormone homeostasis and it is also predominantly involved in detoxification of xenobiotics and therapeutic drugs in the liver^28^. The blood–brain barrier (BBB) and the blood–CSF barrier regulate the composition of the brain milieu. Through structural modifications, including the expression of tight junctions, these CNS barriers restrict the diffusion of drugs and xenobiotics into the brain^29,30^. Aging is an important factor leading to alterations in the biotransformation, either by reduced expression or decreased function and age-associated changes in P450. Corresponding nuclear factors are a major determinant in regulation of drug metabolism, especially during development and in the elderly^29^. As such it is not surprising that we find a large fraction of the age-related metabolites in CSF to be related to the P450 system. We found several substances that directly link to cytochrome P450: dimethyluric acid, barbiturates/pyrimidones, and benzenesulfonamides. Dysfunctional gene expression resulting in changes in mRNA gene regulation has been linked to a destabilization of the cytochrome P450 system with progressing age^11^. This ultimately causes disturbance in cellular energy metabolism, inflammation, and changes in hormone signaling that further destabilize the homeostasis of the human system^28^. In plasma it has mainly been noted that certain xenobiotics (e.g., caffeine) were higher in older subjects, possibly reflecting decreases in hepatic cytochrome P450 activity^9^. We also find metabolites associated with caffeine metabolism such as dimethyluric acid, to be increased in concentration with progressing age. However, the major connections to the P450 system and aging in CSF has, to our knowledge, not previously been demonstrated using this approach.

### Energy metabolism and the immune system

There are clear links between the P450 system, energy metabolism and the immune system. Infection and inflammation, which are managed by the immune system, are also closely related to the P450 system. The abilities of the immune system to generate immune responses decrease gradually with aging leading to an increased susceptibility to infection^31^. The phosphorylated and reduced form of NAD+ , NADPH, serves as a cofactor for P450 enzymes for detoxification for the reduction of oxidized glutathione levels for oxidative defense, and is used for generating peroxides for release during oxidative burst processes in the immune system^32^. Previously, the NAD^+^ pathway has been demonstrated to play a key role in aging^12–14,33^. NAD+ itself has been found to decrease with aging both in the CSF and cells of the CNS^12,34^ while we found increasing levels of NAD+ related metabolites with aging which may indicate dysfunction in the cellular energy metabolism (especially NAD^+^ and adenosine (purine) salvage pathways) and associated perturbations in mitochondrial and ribosomal activity (ubiquinone and guanine pathways)^33,35^. Altered gene regulation with aging and gene defects likely result in the accumulation of catabolites such as dimethyluric acid, hydroxypseudooxynicotine, alpha amino acids, acycloguanosines/hypoxanthines, and fatty alcohols that are normally located in the cytosol^12,13,35^. We also detected compounds which have not been annotated in CSF previously which are related to the ubiquinone pathway (deacetylvindoline, fenpyroximate, and pyrazoles) and are involved in electron transport chain function and influence the production of reactive oxygen species^33^. Taken together, the P450 system, energy metabolism and the immune system play key roles in the process of aging^12^ and metabolites related to these systems also displayed the strongest age association of the CSF metabolites quantified herein.

### Purine and pyrimidine metabolism

Purines, pyrimidines and their intermediates are essential biomolecules that participate in diverse cellular functions, such as the synthesis of DNA, RNA, lipids, and carbohydrates^36^. We found increasing concentrations of purine and pyrimidine related intermediates with aging in human CSF. In mice models of the aging brain, accumulation of purines, pyrimidines and nucleosides have been observed^26^ and decreased levels of pyrimidine intermediates in aged *C. elegans*^37^, suggesting that pyrimidine metabolism is affected in aging. In support of this, supplementation of pyrimidine intermediates can significantly prolong the lifespan of *C. elegans *^38^.

We found a considerable increase in the quantity of 5-hydroxy-l-tryptophan, α,β-didehydrotryptophan, phenyllactic acids, hydantoins in CSF from older subjects that indicate perturbations in tryptophan, phenylalanine and tyrosine metabolisms and ultimately the kynurenine pathway^39^. We have previously found 5-hydroxy-l-tryptophan to be increased in concentration with aging in a different cohort of individuals^18^ and kynurenine has been demonstrated to increase in concentration with age by others^17^. Deficiencies in tryptophan degradation lead to inflammation, immune response, excitatory neurotransmission, and have further been linked to several psychiatric health disorders like depression, dementia, and schizophrenia^39,40^. Disturbances in the kynurenine pathway have systemic impacts^39^. The kynurenine pathway and resulting quantity of tryptophan modulate the composition of the microbiome and affects the synthesis of NAD^+^ from tryptophan^12^. Tryptophan and its metabolite kynurenine have more recently gained significant interest as an important factor related to aging that also impacts the P450 system through the aryl hydrocarbon receptor^41^, again indicating the P450 system plays a central role in the process of natural aging in humans.

### Methodological aspects and limitations of the study

One of the major challenges in metabolomics studies is metabolite identification. In this study, we combined untargeted metabolomics measurements, in silico chemical classification and identification with semantic annotation. The majority of our annotations (see Table 1) reach level 2 according to the 2007 MSI guidelines^58^. In summary, we confirmed 4 compounds with our in-house library (conforming to MSI level 1 annotation), we matched 32 compounds in public libraries (conforming to MSI level 2 annotation), we annotated 12 compounds using SIRIUS and MetFrag (MSI level 3 annotation) and 8 compounds using our classification framework (MSI level 4 annotation). Three compounds were annotated with a sum formula and 10 metabolite features could not be annotated. This top-down methodological approach allowed us to describe the major effects of aging on CSF metabolites in humans and link them mechanistically and functionally to pathways and diseases at a global level. For classification, we performed in silico classification with the MetFamily classifier in positive and negative ion modes^42^. The classifier was trained with ~ 57,000 spectra from the MassBank of North America (MONA) which cover the majority of metabolites that can be expected in CSF^43^.

We applied our classifier to the selected metabolite features that have a high impact on aging. Our classifier works on any kind of MS2 spectrum^44^. For example, our classifier annotated many chemical entities belonging to the classes of purines and pyrimidines. They are involved in DNA repair as they are the building blocks of nucleic acids. Applying functional semantic analysis, we found an increase in the abundance of these compounds with advanced chronological age which may indicate increased expression of genes that mediate stress responses and repair damaged DNA^45^. Similarly, classification found many entities belonging to the classes of tryptophan, phenylalanine, tyrosine, and guanidines without the need to explicitly identify or elucidate the structure of these unknown compounds. The presented methodological approach allows for a systemic view of aging and is suited to identify broad molecular patterns and metabolic pathways where flux may be altered^42^. Using CSF as an explorative source, further research is needed to elucidate the individual mechanistic components, i.e., the compounds that play a causative role in organismal aging^44^.

We conclude that the molecular processes of advanced chronological aging are caused by the complex interplay of alterations in various pathways which degrade immune function and may lead to an increase in adverse microbial catabolites with advancing age. We found the cytochrome P450 system to have a central role in the process of natural aging in humans. Dysfunctional gene regulation has been linked to a destabilization of the cytochrome P450 system with progressing age and ultimately causes disturbance in the cellular energy metabolism, inflammation, and changes in hormone signaling that further destabilize the homeostasis of the human system. Conversely, we found increasing levels of NAD+ with advancing age which accounts for decreased levels of detoxification. Tryptophan-derived degradation products of microbial origin in CSF and tryptophan and its metabolite kynurenine also impact the cytochrome P450 system through the aryl hydrocarbon receptor 40, again indicating the P450 system to be of central role in the process of natural aging in humans. Deficiencies in tryptophan degradation may lead to inflammation, immune response and excitatory neurotransmission which may be associated with neurological disorders like depression, dementia, and schizophrenia^39^. Taken together, this explains why the majority of metabolites increased with advancing age. We determined aging-induced changes in the CSF metabolome. Some of these changes in metabolite abundance may later be shown to be biomarkers for increased risk of neurodegenerative disorders such as Alzheimer’s disease, as brain aging is the largest risk factor for several neurodegenerative disorders. Treatments that delay the appearance of these biomarkers would therefore be promising therapies to delay neurodegenerative disease.

## Methods

### Sampling

Samples were collected by lumbar punctures in routine health care and stored in accordance with the guidelines formed by the BioMS-eu network^46^. Enrollment of study participants, clinical assessment and CSF handling of samples are further described in^47^). The samples were centrifuged at 250 *g* for 5 min and stored in polypropylene tubes in aliquots of 240 µL at − 80 °C until analyzed. We used samples from a control group of subjects that entered the clinic for some reason to have their CSF sampled. We removed one outlier sample for clinical reasons that this subject may have had an undiagnosed neurological disease at the time of sampling.

### Metabolite extraction and mass spectrometry

A detailed description of the metabolite extraction and mass spectrometry analysis is described in^47^. In brief, samples were thawed on ice and 100 µL was mixed with ice-cold methanol spiked with a cocktail of internal standards. After vortexing and 30 min incubation in − 20 °C followed by centrifugation in 12 min at 4 °C, the samples were dried down and reconstituted in 100 µL 5% MeOH, 0.1% formic acid and 94.9% deionized MilliQ water upon analysis. 10 µL was injected in a randomized order constrained to the factor age into a Thermo Ultimate 3000 HPLC equipped with a Thermo Accucore aQ RP C18 column (100 × 2.1 mm, 2.6 µm particle size) and coupled to a Thermo Q-Exactive Orbitrap. A global pool of all samples was injected repeatedly, followed by a blank injection for quality control and filtering purposes. Finally, a twofold serial dilution series ranging from 0.5 to 32.0 µL QC was injected.

### Data analysis

Raw data were converted to mzML-format^48^ and data have been deposited in MetaboLights^49^ under the study identifier MTBLS749. Peak picking was performed using *msconvert* from ProteoWizard^50^ and preprocessed using an OpenMS pipeline in the KNIME platform^51^. Quantification was done using *FeatureFinderMetabo*^52^ and the acquired features were aligned using *MapAlignerPoseClustering* and linked across samples using *FeatureLinkerUnlabelledQT*^53^.

The quantified data from positive and negative modes were separately processed further in the statistical software environment R^54^. Features with a median intensity across the blank injections higher than 1% of the maximum intensity of the samples were interpreted as contaminants and removed. In a second removal step, only features with a significant Pearson correlation (p-value < 0.05) in the dilution series to the injection volumes were kept. To correct for potential run order effects, LOESS curves were fitted for each metabolite using the function *loessFit* from the R package limma and a span of 0.2 was used for normalization. In order to retain only highly stable features, features with a coefficient of variance lower than 20% in the QC samples were kept. The data matrix (positive and negative mod combined) is available as hd_controls_data.csv in the supplemental material.

### Statistics

The statistical analyses comprise the regression of metabolome and age (Random Forest), feature selection (Boruta) and visualization of the high-dimensional data (dbRDA and heatmap). The data and code to reproduce all graphics in this manuscript are provided as supplemental material. Data were normalized prior to statistical analyses, see^47^ for details. Selection of variables that contribute significantly to the effect of aging was accomplished with applying the Boruta algorithm on a Random Forest prediction model^22,55^. Boruta eliminates irrelevant variables by performing permutation tests and comparing the variable importance of features with a background dataset of random features^22^. A Bonferroni post-hoc multiple comparisons adjustment was applied in Boruta on the Random Forest model (using the R package *ranger* and default settings). The following arguments were passed to the function Boruta: x = feature_matrix, y = age, mcAdj = TRUE, maxRuns = 1000, doTrace = 0, holdHistory = TRUE, getImp = getImpRfZ. Although Boruta already calculates out-of-bag (OOB) errors internally and validates selected variables according to paired t-tests^22^, to validate the final model only containing selected variables, we additionally built a regression tree using the function *rtree*. R^2^ was calculated comparing the actual with the predicted model^56^. The above approach was implemented for the full metabolite data in positive and negative modes separately and for the matrix containing selected variables in positive and negative modes. On each of the selected variables, a linear regression was performed with regard to age to determine whether abundances of selected variables were increasing or decreasing with age significantly. A *p*-value < 0.05 was chosen as a significant measure of the linear regression model (Table 1).

To visualize relationships, a heatmap was implemented using the function *heatmap.2* in R. Columns were clustered using a Bray–Curtis dissimilarity measure (implemented in the *vegdist* function of the R package vegan^57^). For the rows, a Euclidean dissimilarity measure was chosen. Both columns and rows were agglomerated using the *Ward.D* method. The two topmost nodes within the “old age” group and the third and second of the second topmost nodes within the “middle age” group were swapped using the function *reorder* of the R package *dendextend* for improved visual representation.

Distance-based ReDundancy Analysis (dbRDA) was performed using the R package *vegan* to determine the effect and direction of aging on the metabolite profiling data^21^. Using the function *envfit*, the factor age was then fitted post-hoc on the dbRDA model. This approach was carried out for the metabolite matrices in positive and negative ionization modes and for the matrix with only the selected features separately (see below on variable selection). The goodness of fit statistics were assessed on the models using the squared correlation coefficient r^2^. Empirical p-values for the factor age were calculated *post-hoc* with *envfit* using permutation.

### Identification and in silico chemical classification

Annotation of selected metabolite features was first accomplished by searching for matching spectra in our in-house library, which resulted in four hits (features pos568, pos726, neg301, neg461). This corresponds to level 1 annotation according to the 2007 Metabolomics Standards Initiative (MSI) guidelines^58^. 32 compounds were matched with public libraries (MSI level 2 annotation). In order to improve the identification of unknowns, annotation was additionally carried out with msPurity^23^ using the MetFrag and SIRIUS Galaxy modules, including the spectra in our in-house library. With this procedure, we followed the level 3 identification in terms of the MSI recommendation for metabolite identification reporting^58^. KEGG and PubChem were chosen as databases in the MetFrag module and BioCyc in the SIRIUS module^24^. Annotation was carried out for positive and negative modes separately. The following parameters were chosen for the SIRIUS module of msPurity: Database: BioCyc, Mass deviation of the fragment peaks in ppm: 10, Maximum number of candidates in the output: 50, Ion mode: positive or negative accordingly, Analysis used: Orbitrap, Schema: Auto, Minimum number of MS/MS peaks: 1. The following parameters were chosen for the MetFrag module of msPurity: Compound Database: PubChem and KEGG separately, Relative Mass Deviation for database search (ppm): 10, Fragment Peak Match Relative Mass Deviation (ppm): 5, Fragment Peak Match Absolute Mass Deviation (Da): 0.01, Polarity: positive or negative accordingly, Schema: Auto, MetFrag Score Types: FragmenterScore OfflineMetFusionScore SuspectListScore, MetFrag Score Weights: 1.0,1.0,1.0. Using this approach, we were able to annotate additional 19 compounds (MSI level 3 annotation).

The chemical classification was performed in silico^42^. In short, the resulting classifier was trained using a machine learning approach on a set of ~ 57,000 MS2 spectra with known structures from the MassBank of North America (MONA) enriched with terms from the ChemOnt ontology^59^. Area under the precision-recall curves (AUC-PR) and true positive for a fixed false negative rates of 5% (TPR-FNR) were calculated for each class separately to assess the correctness of the classifier (Table S1). We used our in-house library containing 451,301 spectra occurring in CSF to extract primary chemical classes using ClassyFire^59^. This resulted in a set of 190 predefined chemical classes that we have previously found in CSF (Table S1, S2).

Then, we classified spectra on the entire set of MS2 DDA spectra in positive and negative mode and on the selected 69 features separately. We used the following settings: absolute mass error: 0.01 Da, ppm error: 10, retention time error: 10 s; for merging similar spectra: retention time range: 20 s, *m/z* range: 0.01 Da, minimum retention time: 10 s, maximum retention time: 1020 s, minimum *m/z*: 50, Maximum *m/z*: 1500, intensity cut-off: 10. Then, the spectra were run through our classifier using the following settings: minimum intensity of maximal MS2 peak: 10, minimum proportion of MS2 peaks: 0.005, *m/z* deviation for absolute grouping: 0.01, *mz* deviation in ppm for grouping: 10, maximum retention time difference: 20 s, *m/z* deviation for absolute mapping: 0.01.

Applying the classifier to the MS2 spectra resulted in sets with scores for one primary (parent) class and alternative parents (ancestors) based on the reference set of 190 chemical classes. Sunburst plots showing the hierarchy and richness of compound classes were constructed using the methodology presented in^42^. The differences between the entire set of MS2 spectra and the set of 69 selected features were determined using Fisher’s exact test (choosing a conservative p-value of 0.005 and choosing only enriched compound classes with a difference greater than zero). The conservative p-value resulted in 45 enriched compound classes which are shown in Fig. 3c. The open-source software inkscape (https://inkscape.org) was used to add figure labels.

### Functional semantic classification

Semantic classification was performed to explore functional relationships of metabolites and compound classes in the human body. The web-APIs of HMDB, PubChem, ChEBI, KEGG and BioCyc were queried on the identifiers of the annotated compounds and classes. The ontology information of the associated semantic metadata was extracted from the resulting XML objects. As ontology information was extracted from different data sources, ontology terms with different order and rank were sorted, corrected for notation and case, and combined prior to subsequent functional annotation. The following ontologies were removed from the dataset: Target-based classification of drugs, Anatomical Therapeutic Chemical, Major components of natural products, every ontology beginning with Disposition/Pathways/SMP and Disposition/Pathways/map, Disposition/Source, Process, Physiological effect, Disposition/Route of exposure, Disposition/Cellular Location, Disposition, Taxonomy, and superclasses without entities: Metabolic pathways, Role, Role/Industrial application, Role/Industrial application/Pharmaceutical industry, Biological location/Biofluid and excreta, and Biological location/Subcellular.

To show the functional semantic entities associated with the annotated metabolites, ontologies were converted to a dendrogram object in R followed by constructing a treemap with the combined ontologies using the *treemap* R package^60^. Similarly, the packages ape, data.tree, and plyr were used to construct a circular tree. Inkscape was used to rearrange text in the plots and to add figure labels.

### Compliance with ethical standards and patient consent statement

The study was approved by the Regional Ethical Review Board in Uppsala, Sweden (Dnr 2012/274 and 2013/278). All participants provided written informed consent before any samples were collected.

## Supplementary Information

Supplementary Information.

## Data Availability

All plots, the required data objects, and the code to fully reproduce the results have been made available in the public repository Zenodo (doi:10.5281/zenodo.5082928). The metabolomics raw data is available in the public MetaboLights data repository^61^ with the identifier MTBLS749.
