# Supplementary material for: Metabolic drift in the aging nervous system is reflected in human cerebrospinal fluid
Source: Sci Rep. 2021 Sep 22;11:18822. doi: 10.1038/s41598-021-97491-1 (PMC8458502; doi:10.1038/s41598-021-97491-1)
Supplement: Supplementary file 1 — Supplementary Information. [file 41598_2021_97491_MOESM1_ESM.pdf]

Supplemental Information

Dynamic Time Warping time series clustering

Methods: In order to determine the correlation type (whether the selected features were linearly or non-linearly correlated with age), a Dynamic Time Warping (DTW) time series clustering was performed [1]. To this end, each feature was fitted into a loess curve (Local Polynomial Regression Fitting) using the *loess* function in R. Time series clustering was performed on the resulting z-normalized loess curves using the function *zscore* and *tsclust* of the package dtwclust. A total of 6 desired clusters were chosen using a hierarchical clustering method, centroid shape extraction and DTW distance measure.

Results: The majority of the selected features were linearly correlated with age. We detected 49 features that were increasing linearly with age (Fig. S1 Type 2), 12 features that were partially linearly correlated with age until the end of the group “middle age” (Fig. S1 Type 3) and 4 features (neg5, neg227, neg311, neg441) that were decreasing almost linearly with age (Fig. S1 Type 5). The feature neg446 had a sinus-like correlation with age (Fig. S1 Type 6) with the lowest abundance in the group “old age”. These features were also identified as decreasing by the heatmap (Fig. 2). The two features pos1168 and pos928 had a quadratic-like correlation (Fig. S1 Type 1) and the feature pos697 had a negatively quadratic-like correlation with age (Fig. S1 Type 4).

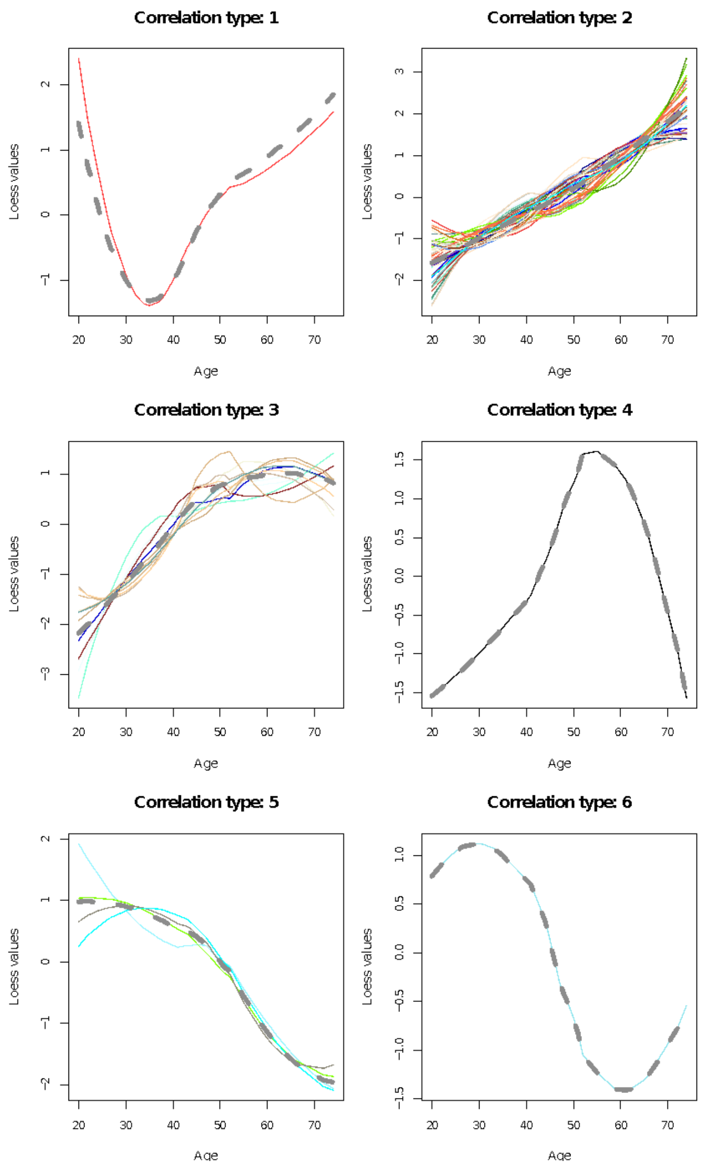

Figure S1: Six types of linear and non-linear correlations in the selected metabolite features for both positive and negative ion mode as determined with Dynamic Time Warping (DTW) time series clustering. The x-axis shows the age and the y-axis shows the normalized loess fitted intensity curves of the corresponding metabolite features. The thin colored lines represent features that were categorized by DTW in the corresponding correlation type. The thick dashed grey line in each subplot represents the average. Type 1: n=2. Type 2: n=49. Type 3: n=12. Type 4: n=1. Type 5: n=4. Type 6: n=1. Total: n=69. Hierarchical clustering distance measures: Type 1: 5.849. Type 2: 4.807. Type 3: 5.874. Type 4: <0.001. Type 5: 4.121. Type 6: <0.001.

Linear regression of selected variables

Variables (metabolite features) were selected using the Boruta algorithm using a Random Forest prediction model (refer methods section on statistics). The selected features were additionally tested for linear regression with age using a linear model. Figure S2 shows plots of the regression for each of the selected metabolite features.

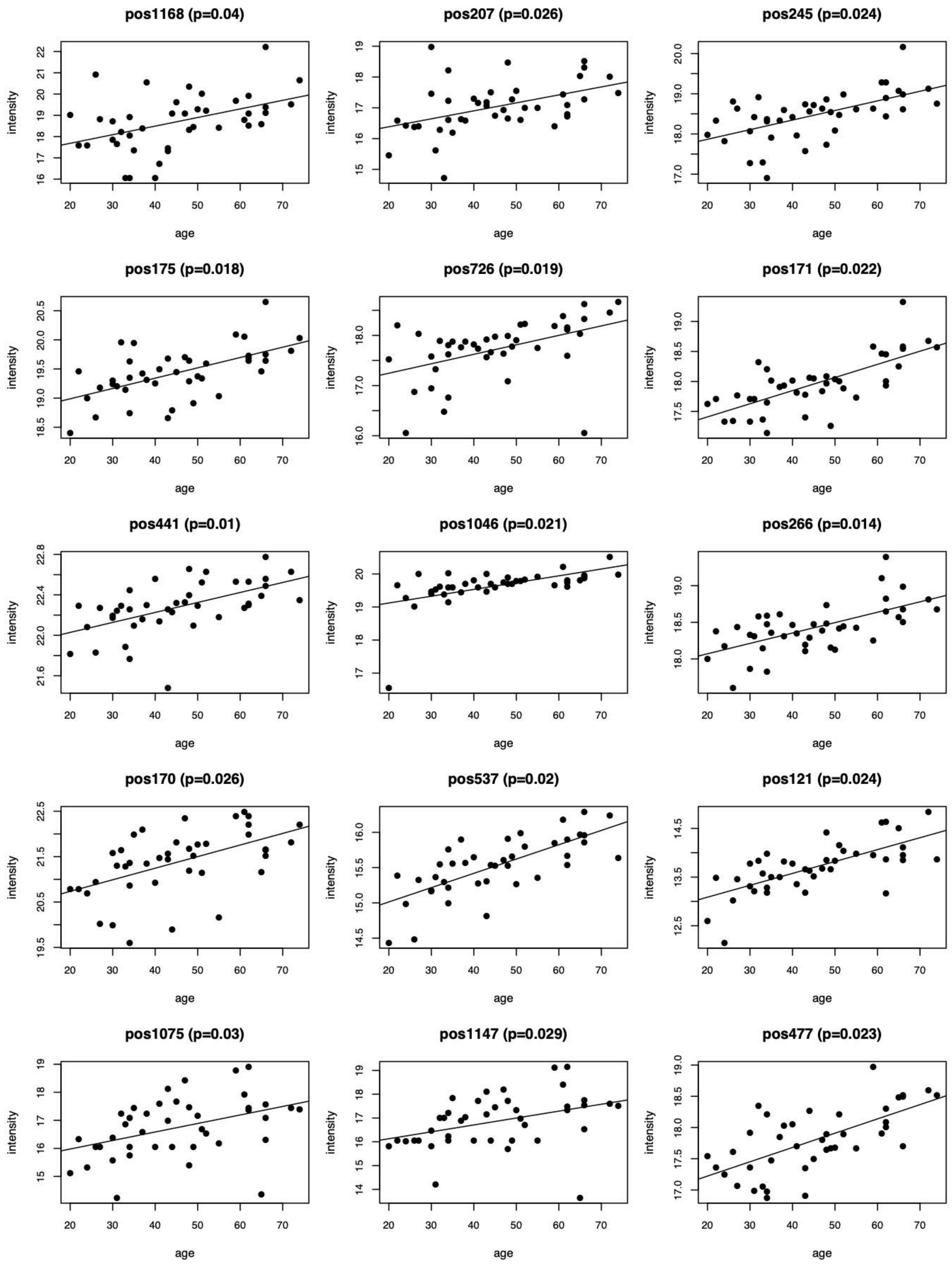
pos1168 (p=0.04)
pos207 (p=0.026)
pos245 (p=0.024)
pos175 (p=0.018)
pos726 (p=0.019)
pos171 (p=0.022)
pos441 (p=0.01)
pos1046 (p=0.021)
pos266 (p=0.014)
pos170 (p=0.026)
pos537 (p=0.02)
pos121 (p=0.024)
pos1075 (p=0.03)
pos1147 (p=0.029)
pos477 (p=0.023)
intensity
age

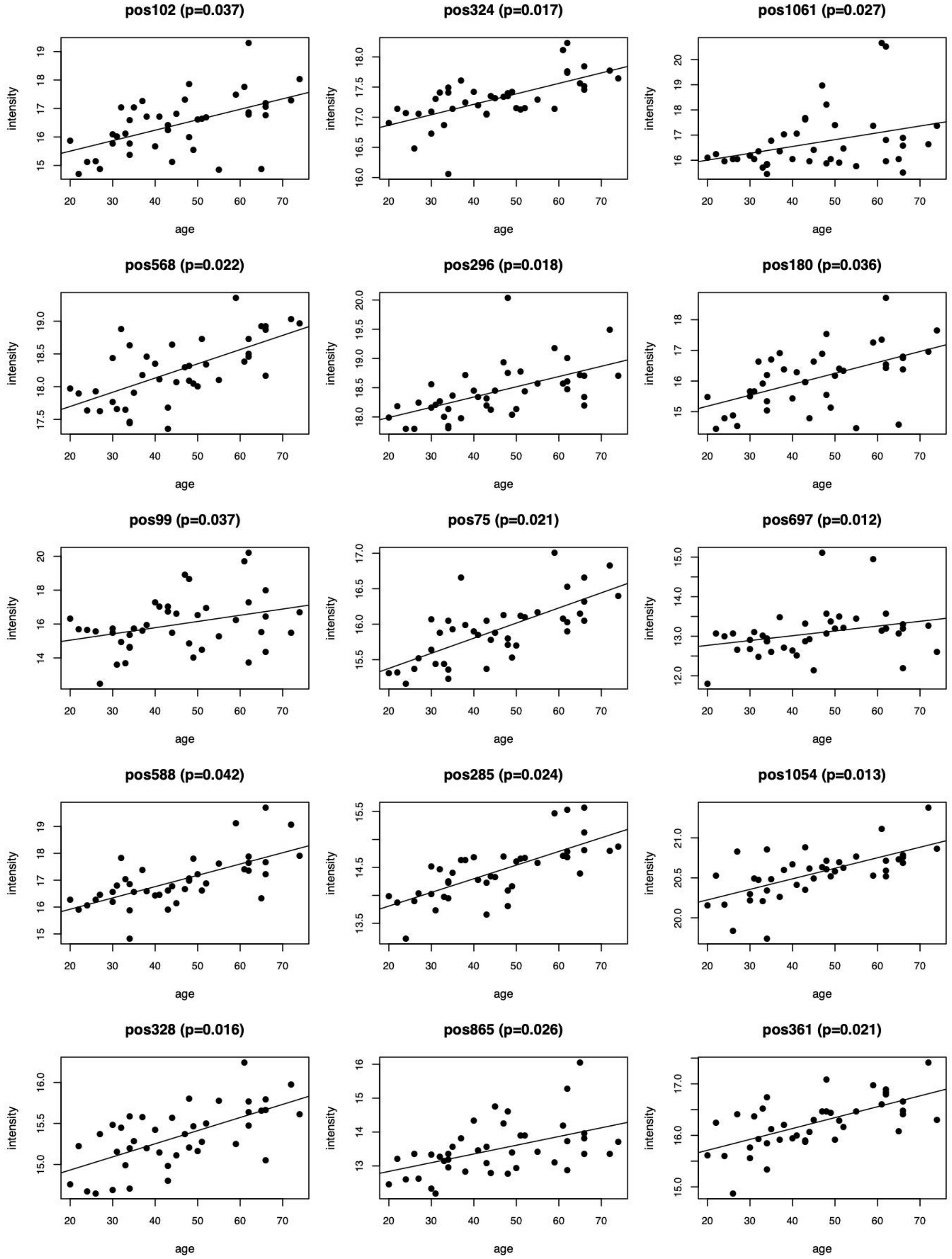
pos102 (p=0.037)
pos324 (p=0.017)
pos1061 (p=0.027)
pos568 (p=0.022)
pos296 (p=0.018)
pos180 (p=0.036)
pos99 (p=0.037)
pos75 (p=0.021)
pos697 (p=0.012)
pos588 (p=0.042)
pos285 (p=0.024)
pos1054 (p=0.013)
pos328 (p=0.016)
pos865 (p=0.026)
pos361 (p=0.021)
intensity
age

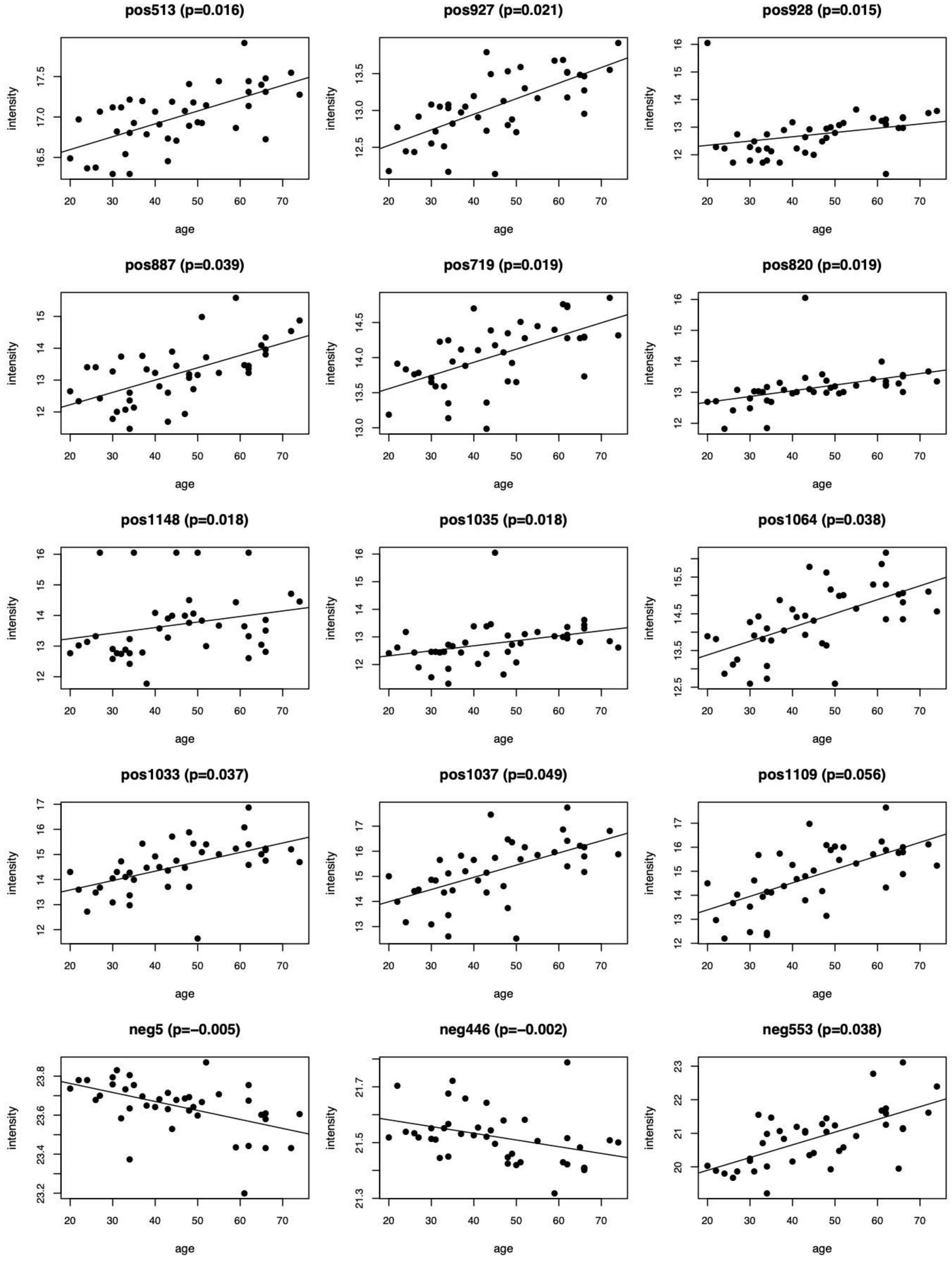
pos513 (p=0.016)
pos927 (p=0.021)
pos928 (p=0.015)
pos887 (p=0.039)
pos719 (p=0.019)
pos820 (p=0.019)
pos1148 (p=0.018)
pos1035 (p=0.018)
pos1064 (p=0.038)
pos1033 (p=0.037)
pos1037 (p=0.049)
pos1109 (p=0.056)
neg5 (p=–0.005)
neg446 (p=–0.002)
neg553 (p=0.038)
intensity
age

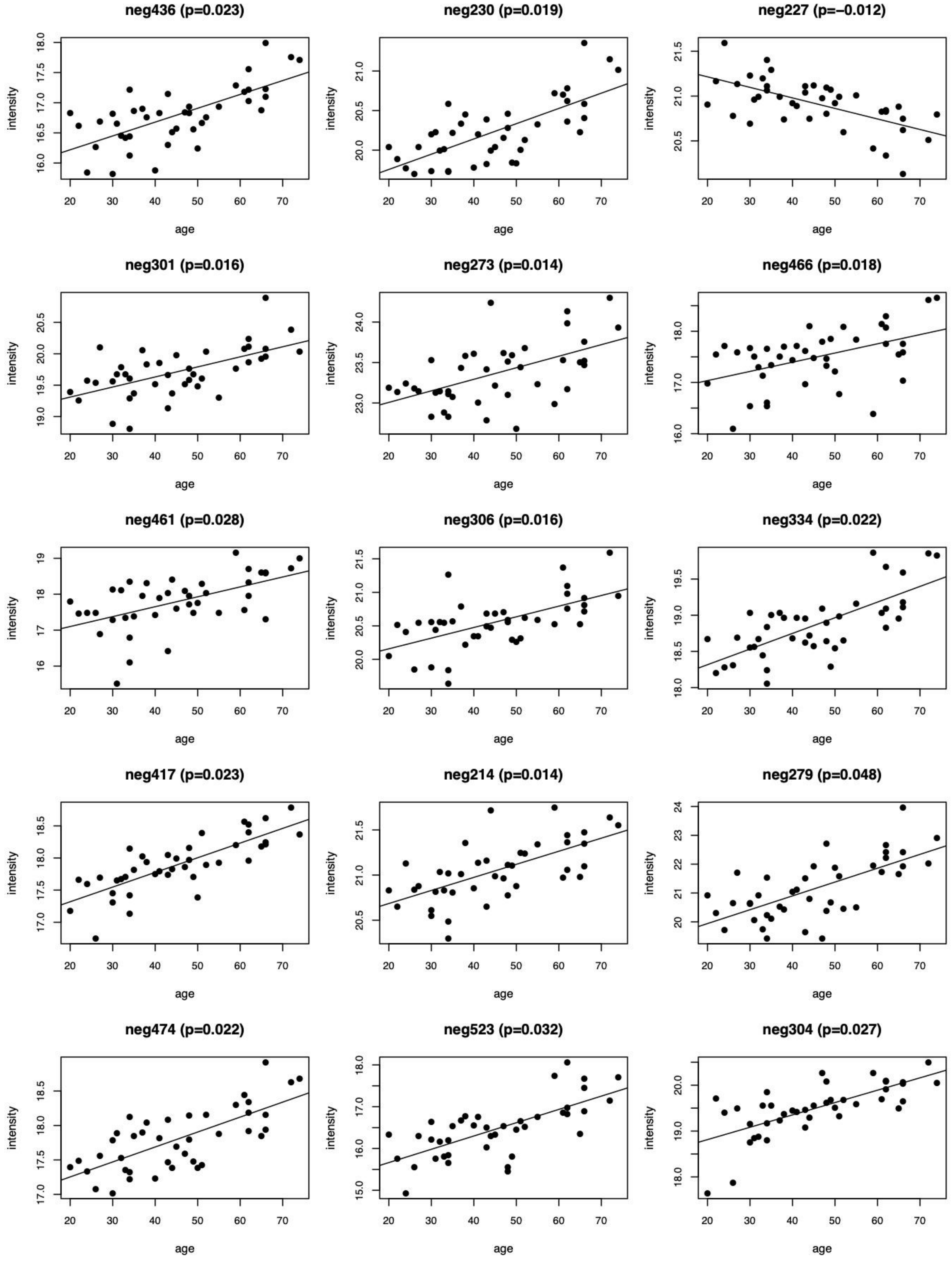
neg436 (p=0.023)
neg230 (p=0.019)
neg227 (p=−0.012)
neg301 (p=0.016)
neg273 (p=0.014)
neg466 (p=0.018)
neg461 (p=0.028)
neg306 (p=0.016)
neg334 (p=0.022)
neg417 (p=0.023)
neg214 (p=0.014)
neg279 (p=0.048)
neg474 (p=0.022)
neg523 (p=0.032)
neg304 (p=0.027)
intensity
age

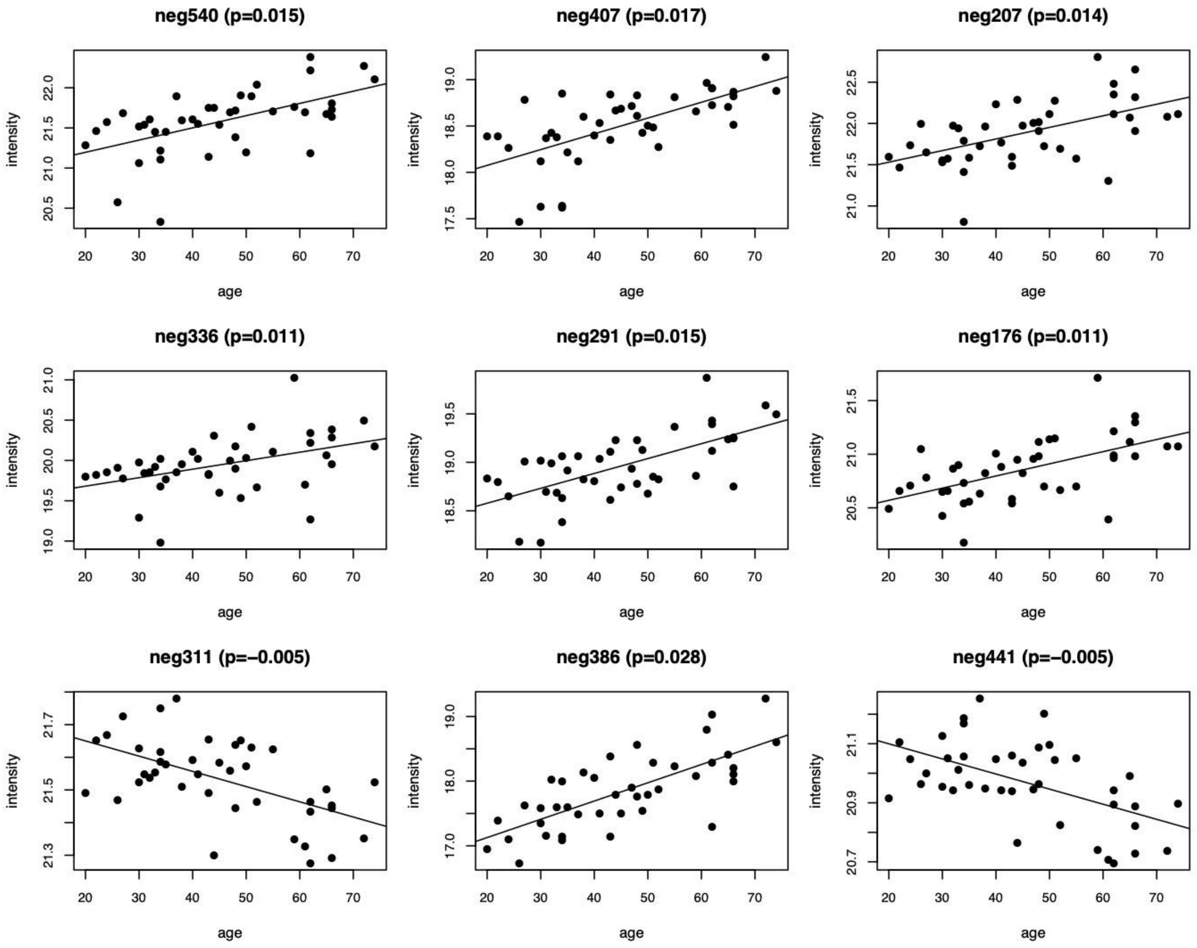

Figure S2: Linear regression of abundances of the 69 metabolite features as selected by the Boruta model (using same ID as Table 1). The x-axis of the plot shows the age of the subject, the y-axis the intensity (TIC) of the metabolite feature. Shown in brackets is the p-value of the linear model. *p*-values below 0.05 indicate significant relationships, positive *p*-values indicate increase with age, while negative *p*-values show decrease with age.

Compound classification and validation

Compound classification was performed in Galaxy using modules of MetFamily (https://github.com/korseby/MFam-autoclass).

Table S1: Number of spectra that were assigned to compound classes in positive ion mode and negative ion mode. Features belonging to primary and secondary classes (parent and ancestor classes) were counted multiple times in case they were detected by *in silico* classification.

Compound class	CHEMONT-ID	Pos-mode	Neg-mode
Alkaloids and derivatives	0000279	1	7
Benzenoids	0002448	46	111
Anthracenes	0000018	0	5
Benzene and substituted derivatives	0002279	18	71
Aniline and substituted anilines	0000284	3	13
Benzenesulfonic acids and derivatives	0000032	0	9
Benzoic acids and derivatives	0000176	6	12
Benzoyl derivatives	0000321	5	13
Phenethylamines	0000186	1	9
Phenylmethylamines	0000185	2	0
Naphthalenes	0000023	2	6
Phenols	0000134	23	14
Benzenediols	0001286	10	1
Methoxyphenols	0000190	4	4
Lipids and lipid-like molecules	0000012	41	105
Fatty Acyls	0003909	20	45
Fatty acid esters	0000324	7	7
Fatty acids and conjugates	0000262	3	19
Lineolic acids and derivatives	0000504	0	9
Glycerolipids	0000175	0	2
Glycerophospholipids	0000256	0	16
Glycerophosphocholines	0002213	0	5
Glycerophosphoethanolamines	0002215	0	4
Prenol lipids	0000259	14	7
Diterpenoids	0001551	7	0
Monoterpenoids	0001549	1	0
Steroids and steroid derivatives	0000258	3	21
Hydroxysteroids	0001295	1	10
Pregnane steroids	0003569	1	0
Nucleosides, nucleotides, and analogues	0000289	41	31
Purine nucleosides	0000479	13	6
Purine ribonucleotides	0001544	12	6
Pyrimidine nucleosides	0000480	0	7
Pyrimidine ribonucleotides	0002147	0	4
Organic acids and derivatives	0000264	165	242
Carboximidic acids and derivatives	0002285	20	22
Carboximidic acids	0002484	10	11
Carboxylic acids and derivatives	0000265	35	92
Amino acids, peptides, and analogues	0000013	16	26
Carboxylic acid derivatives	0001093	2	11
Carboxylic acids	0001205	7	19
Dicarboxylic acids and derivatives	0000346	6	5
Peptides	0000348	7	12

Tricarboxylic acids and derivatives	0001986	0	12
Hydroxy acids and derivatives	0000472	13	54
Alpha hydroxy acids and derivatives	0001359	0	20
Beta hydroxy acids and derivatives	0001713	3	17
Organic carbonic acids and derivatives	0000364	39	11
Ureas	0000517	21	5
Organic phosphoric acids and derivatives	0000402	32	19
Phosphate esters	0000408	16	9
Organic sulfonic acids and derivatives	0004434	11	26
Organosulfonic acids and derivatives	0000270	6	13
Peptidomimetics	0001813	2	0
Organic nitrogen compounds	0004707	57	67
Organonitrogen compounds	0000278	41	49
Amines	0002449	2	11
Guanidines	0000375	11	13
Quaternary ammonium salts	0000503	12	8
Organic oxygen compounds	0004603	110	126
Organic oxoanionic compounds	0000463	8	10
Organic pyrophosphates	0001804	4	5
Organooxygen compounds	0000323	89	92
Alcohols and polyols	0000129	10	13
Carbohydrates and carbohydrate conjugates	0000011	57	23
Disaccharides	0001542	10	0
Glycosyl compounds	0002105	16	7
Monosaccharides	0001540	13	7
Carbonyl compounds	0001831	2	21
Ethers	0000254	10	10
Organoheterocyclic compounds	0000002	151	172
Azoles	0000436	35	27
Imidazoles	0000078	16	7
Pyrazoles	0000087	2	9
Benzimidazoles	0000294	3	9
Diazines	0001346	31	16
Pyrazines	0000067	4	0
Pyrimidines and pyrimidine derivatives	0000075	12	8
Imidazopyrimidines	0001797	31	14
Purines and purine derivatives	0000245	16	7
Indoles and derivatives	0000211	0	26
Indoles	0002497	0	13
Lactones	0000050	2	8
Pyridines and derivatives	0000089	18	24
Hydropyridines	0002224	8	0
Pyridinecarboxylic acids and derivatives	0001322	5	12
Pyrroles	0000090	0	13

Pyrrolidines	0000218	8	9
Pyrrolidones	0001158	7	0
Quinolines and derivatives	0001253	3	10
Quinoline carboxylic acids	0002552	2	0
Tetrahydroisoquinolines	0002955	2	0
Thioethers	0001202	24	35
Dialkylthioethers	0003862	11	17
Phenylpropanoids and polyketides	0000261	43	32
Cinnamic acids and derivatives	0000476	18	8
Hydroxycinnamic acids and derivatives	0001391	9	4
Flavonoids	0000334	10	5
Phenylpropanoic acids	0002551	0	11
Stilbenes	0000253	5	0

Table S2: Validation measures of the *in silico* classification shown for each of the detected compound classes acquired in positive and negative ion mode separately. AUC-PR: Area under precision-recall curve. TPR-FPR: true positive rate for a fixed false negative rate of 5%. Values above 0.5 are generally considered good performance metrics. The calculation is explained in [2].

		Positive ion mode		Negative ion mode	
**Compound class**	**CHEMONT-ID**	**AUC-PR**	**AUC-PR**	**TPR-FPR**	**TPR-FPR**
Alkaloids and derivatives	0000279	0,726	0,903	0,214	0,500
Benzenoids	0002448	0,711	0,855	0,152	0,464
Anthracenes	0000018	0,597	0,936	0,200	0,600
Benzene and substituted derivatives	0002279	0,660	0,809	0,073	0,387
Aniline and substituted anilines	0000284	0,714	0,904	0,250	0,600
Benzenesulfonic acids and derivatives	0000032	-	0,825	-	0,600
Benzoic acids and derivatives	0000176	0,632	0,784	0,097	0,188
Benzoyl derivatives	0000321	0,591	0,762	0,059	0,161
Phenethylamines	0000186	0,714	0,848	0,077	0,400
Phenylmethylamines	0000185	0,370	-	0,000	-
Naphthalenes	0000023	0,622	0,747	0,118	0,143
Phenols	0000134	0,719	0,846	0,158	0,489

Benzenediols	0001286	0,768	0,919	0,211	0,639
Methoxyphenols	0000190	0,718	0,910	0,071	0,500
Lipids and lipid-like molecules	0000012	0,618	0,880	0,243	0,690
Fatty Acyls	0003909	0,645	0,874	0,163	0,678
Fatty acid esters	0000324	0,578	0,972	0,056	0,899
Fatty acids and conjugates	0000262	0,760	0,801	0,214	0,360
Lineolic acids and derivatives	0000504	-	0,878	-	0,600
Glycerolipids	0000175	-	0,932	-	0,556
Glycerophospholipids	0000256	-	0,984	-	0,952
Glycerophosphocholines	0002213	-	0,981	-	0,946
Glycerophosphoethanolamines	0002215	-	0,980	-	0,948
Prenol lipids	0000259	0,548	0,523	0,115	0,143
Diterpenoids	0001551	0,506	-	0,000	-
Monoterpenoids	0001549	0,525	-	0,000	-
Steroids and steroid derivatives	0000258	0,791	0,780	0,556	0,400
Hydroxysteroids	0001295	0,859	0,810	0,542	0,250
Pregnane steroids	0003569	0,891	-	0,667	-
Nucleosides, nucleotides, and analogues	0000289	0,727	0,957	0,194	0,762
Purine nucleosides	0000479	0,795	0,995	0,300	1,000
Purine ribonucleotides	0001544	0,727	0,997	0,375	1,000
Pyrimidine nucleosides	0000480	-	0,939	-	0,625
Pyrimidine ribonucleotides	0002147	-	0,997	-	1,000
Organic acids and derivatives	0000264	0,599	0,807	0,085	0,296
Carboximidic acids and derivatives	0002285	0,622	0,763	0,071	0,200
Carboximidic acids	0002484	0,664	0,766	0,088	0,167
Carboxylic acids and derivatives	0000265	0,612	0,847	0,099	0,483
Amino acids, peptides, and analogues	0000013	0,650	0,851	0,130	0,474
Carboxylic acid derivatives	0001093	0,622	0,886	0,139	0,678
Carboxylic acids	0001205	0,666	0,864	0,165	0,475
Dicarboxylic acids and derivatives	0000346	0,567	0,882	0,125	0,611
Peptides	0000348	0,613	0,876	0,100	0,333
Tricarboxylic acids and derivatives	0001986	-	0,879	-	0,800
Hydroxy acids and derivatives	0000472	0,506	0,844	0,125	0,357
Alpha hydroxy acids and derivatives	0001359	-	0,893	-	0,333
Beta hydroxy acids and derivatives	0001713	0,593	0,901	0,083	0,438
Organic carbonic acids and derivatives	0000364	0,677	0,755	0,103	0,275
Ureas	0000517	0,631	0,825	0,206	0,286
Organic phosphoric acids and derivatives	0000402	0,690	0,985	0,211	0,955
Phosphate esters	0000408	0,724	0,984	0,167	0,958
Organic sulfonic acids and derivatives	0004434	0,763	0,873	0,310	0,667
Organosulfonic acids and derivatives	0000270	0,778	0,875	0,310	0,611
Peptidomimetics	0001813	0,882	-	0,750	-
Organic nitrogen compounds	0004707	0,725	0,861	0,150	0,484
Organonitrogen compounds	0000278	0,720	0,843	0,183	0,413

Amines	0002449	0,632	0,851	0,120	0,523
Guanidines	0000375	0,788	0,940	0,286	0,625
Quaternary ammonium salts	0000503	0,521	0,978	0,000	0,944
Organic oxygen compounds	0004603	0,665	0,827	0,184	0,625
Organic oxoanionic compounds	0000463	0,812	0,999	0,333	1,000
Organic pyrophosphates	0001804	0,731	1,000	0,333	1,000
Organooxygen compounds	0000323	0,657	0,850	0,150	0,560
Alcohols and polyols	0000129	0,640	0,824	0,140	0,382
Carbohydrates and carbohydrate conjugates	0000011	0,805	0,934	0,403	0,715
Disaccharides	0001542	0,843	0,965	0,357	0,909
Glycosyl compounds	0002105	0,810	0,941	0,443	0,728
Monosaccharides	0001540	0,760	0,923	0,250	0,708
Carbonyl compounds	0001831	0,612	0,814	0,114	0,455
Ethers	0000254	0,676	0,793	0,098	0,409
Organoheterocyclic compounds	0000002	0,669	0,859	0,153	0,429
Azoles	0000436	0,654	0,838	0,146	0,430
Imidazoles	0000078	0,625	0,844	0,108	0,444
Pyrazoles	0000087	0,743	0,894	0,333	0,667
Benzimidazoles	0000294	0,758	0,762	0,188	0,400
Diazines	0001346	0,666	0,872	0,160	0,581
Pyrazines	0000067	0,536	-	0,000	-
Pyrimidines and pyrimidine derivatives	0000075	0,661	0,901	0,146	0,625
Imidazopyrimidines	0001797	0,703	0,959	0,158	0,800
Purines and purine derivatives	0000245	0,730	0,963	0,237	0,800
Indoles and derivatives	0000211	0,674	0,831	0,132	0,250
Indoles	0002497	0,727	0,810	0,125	0,357
Lactones	0000050	0,685	0,902	0,138	0,500
Pyridines and derivatives	0000089	0,618	0,705	0,154	0,176
Hydropyridines	0002224	0,487	-	0,083	-
Pyridinecarboxylic acids and derivatives	0001322	0,773	0,879	0,462	0,250
Pyrroles	0000090	0,679	0,825	0,111	0,375
Pyrrolidines	0000218	0,644	0,758	0,111	0,250
Pyrrolidones	0001158	0,684	-	0,333	-
Quinolines and derivatives	0001253	0,652	0,691	0,214	0,333
Quinoline carboxylic acids	0002552	0,973	-	0,750	-
Tetrahydroisoquinolines	0002955	0,835	-	0,200	-
Thioethers	0001202	0,726	0,765	0,289	0,364
Dialkylthioethers	0003862	0,753	0,835	0,409	0,444
Phenylpropanoids and polyketides	0000261	0,780	0,932	0,245	0,685
Cinnamic acids and derivatives	0000476	0,662	0,848	0,100	0,167
Hydroxycinnamic acids and derivatives	0001391	0,736	0,816	0,125	0,167
Flavonoids	0000334	0,866	0,955	0,479	0,805
Phenylpropanoic acids	0002551	0,581	0,910	0,000	0,500
Stilbenes	0000253	0,868	-	0,500	-

Variable selection of features corresponding to gender

Features that represent different genders were selected with the Boruta algorithm calculated post-hoc on a Random Forest (Arguments to function Boruta: x=feature_matrix, y=gender, pValue=0.01, mcAdj=TRUE, maxRuns=1000, doTrace=0, holdHistory=TRUE, getImp=getImpRfZ). Figure S4 shows the selected features for the genders. We found a total of 41 features that were strongly related to gender. As none of the selected features for gender overlapped with the ones selected for the factor age, the effects of gender and aging can be interpreted als largely independent from each other.

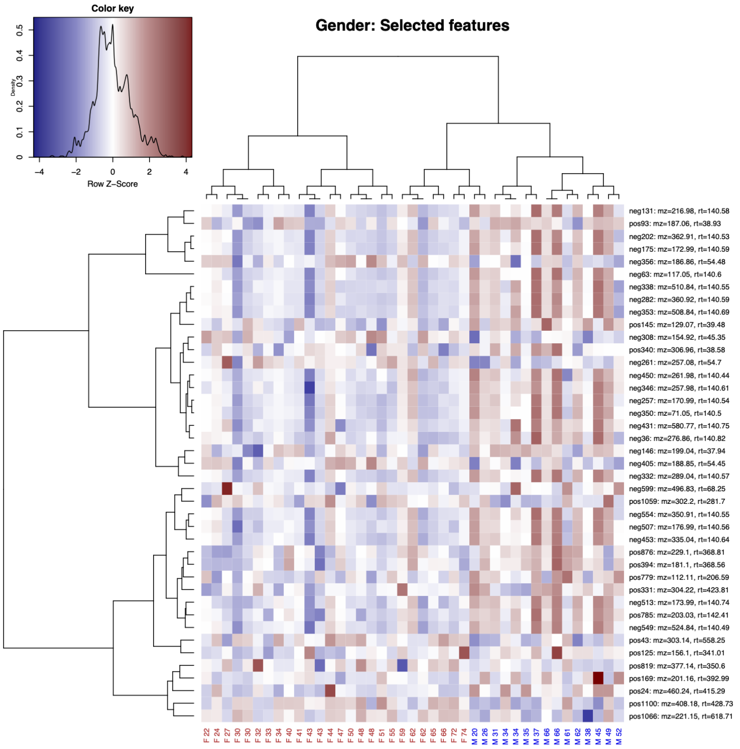

Figure S4: Heatmap showing selected metabolite features clustered in rows and the samples clustered by gender in columns. The first letter of the sample name indicates the gender of the subject (M, blue color: Male, F, red color: Female). A red color in the heatmap indicates an increase in the abundance normalised via the z-score of a respective metabolite feature, a blue color indicates low abundance. R^2_{Boruta}=0.469.

References

1. Giorgino T. Computing and Visualizing Dynamic Time Warping Alignments in R: The dtw Package. J Stat Softw. 2009;031. doi:http://hdl.handle.net/10.

2. Peters K, Treutler H, Döll S, Kindt ASD, Hankemeier T, Neumann S. Chemical Diversity and Classification of Secondary Metabolites in Nine Bryophyte Species. Metabolites. 2019;9. doi:10.3390/metabo9100222
